# Respite and connection: Autistic adults’ reflections upon nature and well-being during the Covid-19 pandemic

**DOI:** 10.1177/13623613231166462

**Published:** 2023-04-27

**Authors:** Samantha Friedman, Roan Noble, Stephanie Archer, Jenny Gibson, Claire Hughes

**Affiliations:** 1 University of Cambridge, UK; 2 Northumbria University, UK; 3 Independent Consultant, UK

**Keywords:** autism, Covid-19 pandemic, nature, stress reduction theory, well-being

## Abstract

The Covid-19 pandemic and associated lockdowns provided opportunities to engage and reconnect with nature, with many people noting associated benefits to well-being. Research from the pandemic period has largely focused on the way neurotypical or general populations experienced nature; less is known about how autistic people used nature to support well-being during this time. In this qualitative survey study of 127 autistic adults in the United Kingdom, we used reflexive thematic analysis of text box responses to develop two themes: *respite in nature* and *connecting amid widespread disconnection*. For some autistic adults during the pandemic, nature provided physical distance from others or from crowded homes, enabling them to use nature to experience relief from stress. In addition, some participants felt more psychologically connected to nature itself during the pandemic, while for others, nature served as a way of connecting with others during a potentially isolating time. These findings have implications for autistic people and their families and carers who may want to seek out nature-based activities to support well-being in the wake of the pandemic.

**Lay abstract:**

The Covid-19 pandemic and associated lockdowns provided opportunities to spend time in nature, with many people reporting that this benefitted their well-being. However, existing research from the pandemic period has focused on the way general populations experienced nature; less is known about how autistic people used nature to support their well-being during the pandemic. We created a survey that invited autistic adults living in the United Kingdom to reply to text box questions. A total of 127 people responded to our survey; we analysed their responses using a method called reflexive thematic analysis and developed themes based on patterns among all the responses. We developed two themes: *respite in nature* and *connecting amid widespread disconnection*. For some autistic adults during the pandemic, nature provided physical distance from others or from crowded homes, which helped them reduce their stress. In addition, some participants felt more psychologically connected to nature itself during the pandemic, while for others, nature served as a way of connecting with others during a potentially isolating time. These findings are important for autistic people and their families and carers who may want to seek out nature-based activities to support well-being in the wake of the pandemic.

## Introduction and literature review

In early 2020, the Covid-19 pandemic and its associated lockdowns caused widespread disruption and disconnection in the United Kingdom and beyond. Spending time in nature was one way that people dealt with the challenges of the pandemic, particularly as people were allowed a once-daily bout of outdoor exercise during even the strictest UK lockdowns (e.g. Brown et al., 2021; Office for National Statistics, 2021; Wright et al., 2022). Time in and near nature has well-evidenced associations with improved well-being in children (McCormick, 2017; Vanaken & Danckaerts, 2018) and adults (Capaldi et al., 2015; McMahan & Estes, 2015). Mental health is one component of well-being (World Health Organization, 2022), and many autistic adults and children experienced an increase in mental health difficulties during the pandemic (National Health Service, 2020; O’Connor et al., 2020). Thus, nature may provide a low-cost method of support for well-being for some autistic people.

Several theoretical frameworks help explain the potential benefits of exposure to nature. One such theory is Ulrich et al.’s (1991) stress reduction theory (SRT), which posits that natural environments and natural stimuli facilitate recovery from stress. According to this theory, natural environments are less stressful than human-made environments because evolution has led humans to process natural stimuli more easily than artificial stimuli. In this work, changes in stress levels are measured using both subjective participant report and physiological markers (e.g. heart rate variability and salivary cortisol). Others (e.g. Robertson & Simmons, 2015) have shown that groups who are more sensitive to various types of stimuli, including autistic people, find natural visual stimuli easier to process than human-made stimuli. In a study of autistic adults’ sensory experiences, MacLennan et al. (2022) reported that many autistic adults seek out natural scenes. However, the authors’ discussion of the varied (and sometimes contradictory) stimuli that can be distressing for autistic adults highlights the heterogeneity of autistic peoples’ experiences with sensory stimuli. In a subsequent study, MacLennan, Woolley et al. (2022) noted that outdoor spaces were among the most enabling sensory spaces, according to autistic participants. Natural stimuli may therefore serve a calming or beneficial purpose for some (but not all) autistic people.

Despite the benefits, little is known about how autistic people experience nature or how nature may be linked to improved well-being in autistic people. Well-being is an area of urgent focus in the autistic community given the mental health crisis occurring in this group (Vasa et al., 2020). The larger autism community has repeatedly called for the prioritisation of well-being-focused research, but progress towards achieving this goal has been slow (Frazier et al., 2018; Roche et al., 2021). One contributor to poor mental health in autistic groups is camouflaging (Cage et al., 2018; Cage, Troxell-Whitman, 2019). To contend with challenging social interactions predicated on neurotypical norms, many autistic people use camouflaging techniques, namely, masking certain traits or behaviours to appear more socially normative (Hull et al., 2017). Beyond the negative impacts of camouflaging, autistic people also experience mental health challenges, including anxiety and depression, at a higher rate than the neurotypical population; Lai et al. (2019) reported a 20% pooled prevalence of anxiety disorders among autistic people, compared with 7.3% in the general population and a 11% pooled prevalence of depressive disorders in autistic people, compared with 4.7% in the general population. Loneliness in autistic adults is a prevalent but under-studied issue that also impacts on well-being (Umagami et al., 2022).

During the Covid-19 pandemic, autistic people’s increased likelihood of experiencing mental health problems remained true; Oomen et al. (2021) compared mental health in autistic and non-autistic adults and reported small to medium effect sizes. However, the pandemic had a varied mental health impact on autistic adults, with some autistic people noting that the pandemic provided a break from sensory overload, fewer unwanted social interactions, less need to camouflage and increased feelings of hopefulness (Bal et al., 2021; Lois Mosquera et al., 2021; Oomen et al., 2021). Despite these silver linings, for many, increased social isolation (Hedley et al., 2021; Pellicano et al., 2021) and disruptions to routine (Bundy et al., 2022; Davidson et al., 2021) were linked to poorer well-being.

Taylor et al. (2021) conducted three studies of links between autistic traits and pro-environmental attitudes and behaviours. Taylor et al. suggest several reasons why autistic traits may relate to these concepts; these include the likelihood of autistic people having focused interests in nature, autistic people disliking change (i.e. climate-related change or environmental degradation) and the prevalence of sensory sensitivities among autistic people that may be supported through nature. Surprisingly, given these reasons and the examples of prominent autistic environmental activists (e.g. Greta Thunberg, Chris Packham), they found that participants’ self-reported autistic traits, as indexed by the short version of the Autism Spectrum Quotient (Allison et al., 2012), were associated with a *decreased* likelihood to enact pro-environmental behaviours. This unexpected result highlights the need to better understand how autistic people experience nature and raises the possibility that neurotypical and autistic individuals differ in how they engage with concepts such as pro-environmental attitudes. Note also that measuring autistic traits may not be an accurate way of representing the experiences of either self or professionally diagnosed autistic people (Sasson & Bottema-Beutel, 2022): above the diagnostic threshold, there may be qualitative differences in how a person experiences these nature-related constructs.

Alongside disruption and challenges, the Covid-19 pandemic gave many people across the world unprecedented opportunities to engage with nature. In the United Kingdom, people took part in outdoor exercise more frequently during the lockdown periods than in pre-pandemic times, and once lockdown ended in summer 2020, there was over a 60% increase in visits to parks and natural areas compared with pre-pandemic years (Office for National Statistics, 2021). Concurrently, however, the amount of sedentary behaviour exhibited by people of all ages, both in the United Kingdom and globally, increased drastically during the pandemic (Kass et al., 2021; Stockwell et al., 2021). As a result, nature experiences during the pandemic were likely to vary with geographic location, personal interests and individual circumstances.

International research has demonstrated that during the pandemic various types of interactions with nature (e.g. viewing nature, spending time in nature or psychological connection to nature) were linked to improved well-being in general population samples of adults (e.g. Soga et al., 2020) and children (e.g. Friedman et al., 2021; Hazlehurst et al., 2022). Compared with these non-autistic samples, autistic people are likely to have experienced similar or worse challenges to well-being during the pandemic, but their experiences of nature or how these experiences may have been linked with autistic people’s well-being has, thus far, received little research attention.

### Research questions

Responding to the opportunity offered by the Covid-19 pandemic to better understand how autistic people experienced nature at a time of significant stress and disruption, our exploratory survey study considered two research questions: What role did nature play in promoting (or diminishing) well-being for autistic adults during the Covid-19 pandemic? How do autistic adults perceive their relationship with nature changed (or did not change) during the Covid-19 pandemic?

## Methods

### Ethical approval and procedures

The University of Cambridge Psychology Research Ethics Committee approved this survey study (reference number PRE.2021.073), which was created, pre-registered on the Open Science Forum repository and stored on the Qualtrics online platform (see Supplemental Appendix 1 for a copy of the survey, including participant information sheet).

### Sampling strategy

Participants were recruited through both convenience (Frey, 2018) and purposeful sampling (Palinkas et al., 2015) via recruitment messages distributed on social media and through newsletters of autistic groups. After several weeks, we noticed a lack of respondents from outside of England and asked colleagues in Northern Ireland, Wales and Scotland to share the recruitment information with their networks. We welcomed participation from autistic adults who were self-diagnosed and those who had a clinical diagnosis.

### Survey methods, questions and community involvement

Survey methods offer an efficient means of conducting accessible qualitative research with autistic people (Crane et al., 2020). This survey was developed in partnership with an autistic adult and piloted by two additional autistic people; all three were paid for their time spent working on the study. Feedback from these autistic consultants shaped what questions were asked in the survey, how they were written and how the survey was formatted. For instance, the primary researcher, S.F., wanted to include a formal measure to ascertain participants’ sensory profiles; however, the primary autistic consultant suggested that it would be best to let participants self-describe their sensory needs. Additionally, the autistic adults who piloted the study pointed out that several questions in the survey were redundant, that the wording of some questions were confusing and needed clarification and that it would be useful to provide examples of potential ways to answer questions. The autistic consultants also provided guidance on how to format pages of the survey (e.g. breaking up the text) to make it easier to read and process. Suggestions from the autistic consultants were applied in almost all cases; the only instances of disagreement occurred when the people piloting the survey had suggestions which contradicted those from the primary autistic consultant. These disagreements were discussed with the primary consultant to arrive at a mutually agreed-upon way forward. The primary autistic consultant is an author on this article and reviewed the work prior to submission.

Guided by suggestions from the autistic consultants, we also included a page at the start of the survey that explained the types of questions included in the survey and defined key terms used throughout to ensure participants were operating on a shared understanding of these terms (see Supplemental Appendix 1). As part of that page, the following information was provided to participants:

For this survey, nature is defined as anything in the physical world including outdoor green spaces, animals, other landscape features like mountains and rivers, and plants.

Spending time or being **IN** nature refers to being outside amongst features of the physical world, including green spaces, animals, and plants. This includes interacting with nature by touching it or using natural materials as well as sitting, walking, or otherwise moving in natural spaces.

### Analysis and rigour

The online survey contained 13 close-ended questions (i.e. nominal and binary questions) and 13 open-ended questions (i.e. text box questions that allowed for multiple sentences of written text). Here, we analyse responses to two open-ended questions that were most relevant to understanding nature’s relationship with well-being during the Covid-19 pandemic:

- For participants who reported a change in their relationships with nature: How did your relationship with nature change because of the Covid-19 pandemic and lockdowns?
- Does being unable to access nature have an impact on your mental health? If so, how?

We analysed the selected open-ended text box questions using Braun and Clarke’s (2006, 2019, 2021) reflexive thematic analysis, which offers both theoretical and methodological flexibility. This flexibility was important as respondents provided differing levels of richness in their responses. Beginning with the question ‘How did your relationship with nature change because of the Covid-19 pandemic and lockdowns?’ (and repeating the process for the second question), the first author (S.F.) printed and read all responses, highlighting words or phrases that stood out and taking notes of initial thoughts in the margins. Next, she re-read the responses and conducted the first round of formal coding before starting a second round, moving through the dataset in the opposite direction.

Next, S.F. compiled all the codes for that question onto a sheet of paper and began grouping related codes by colour, developing candidate themes and descriptions of each group of codes. After repeating this process for both questions, S.F. met with a co-author, S.A., an experienced qualitative researcher who does not have expertise in this specific topic (allowing her to offer a different perspective). S.F. presented the candidate themes to S.A. using thematic maps and explained coding choices. Following suggestions from Braun and Clarke (2021), S.F. and S.A. used peer debriefing and audit trails to enhance the rigour of the qualitative analysis presented here. S.F. then wrote up the findings, a crucial part of the analysis process (Braun & Clarke, 2021), which helped us refine our ideas. When including participant quotes from written survey responses, text is unedited except where spelling or grammar errors impacted readability. Identifiable information has also been removed from responses and replaced with descriptive words.

### Positionality

Within the Qualitative paradigm, the explicit acknowledgement of the positionality of the researchers also increases rigour as the researchers’ experiences and perspectives directly shape the findings they develop (Braun & Clarke, 2021). The primary researcher, S.F., is a non-autistic researcher working in the fields of Education and Psychology. She is also a qualified Level 3 Forest School leader and therefore believes in the potential for nature to support well-being for some people. S.F. strives to align her work with the neurodiversity paradigm (den Houting, 2019), a constructivist epistemology (Constantino, 2008) and a critical realist ontology (Braun & Clarke, 2021).

## Findings

### Participant demographics and descriptive information

Survey completion time varied from 7 min to 2 h. The survey was launched on 28 October 2021 and closed on 25 November 2021. In that time, 127 autistic adults responded. Demographic information of the respondents is provided in Table 1.

**Table 1.** Participant demographics.

	Total participants (*n* = 127)	
	%	*n*
Gender		
Women	60.6	77
Men	26.0	33
Non-binary	10.2	13
Other	3.1	4
Age (years)		
18–24	17.3	22
25–34	18.9	24
35–44	25.2	32
45–54	22.8	29
55–64	12.6	16
65–74	3.1	4
Employment		
Full or part-time	39.4	50
Not employed	10.2	13
Student	14.2	18
Retired	2.4	3
Unable to work	18.9	24
Other	15	19
Location		
England	81.1	103
Scotland	11.0	14
Wales	4.7	6
Northern Ireland	2.4	3
Prefer not to answer	0.8	1
Has a focused interest		
Yes	86.6	110
No	7.9	10
Prefer not to answer	3.9	5
focused interest in nature		
Yes	52.0	66
No	31.5	40

Of the total respondents, 30.4% (*n* = 38) indicated that they had accessibility needs that affect how often they leave home and where they can go. These values align with estimates from a representative Scottish study of over 5000 people, which indicate that between 24% and 42.2% of autistic adults have a physical disability, depending on the presence of co-occurring intellectual disability (Dunn et al., 2020; Rydzewska et al., 2018).

When asked about the impact of the Covid-19 pandemic and related lockdowns on the time they spent in nature, 47.9% of respondents (*n* = 58) reported that they spent more time in nature while 27.3% (*n* = 33) reported that they spent less time in nature and 24.8% (*n* = 30) reported that they spent the same amount of time in nature as usual. Beyond simply spending time in nature, 43% of respondents (*n* = 52) indicated that the Covid-19 pandemic and associated lockdowns also contributed to a change in their relationship with nature.

### Thematic findings

**Figure 1.**
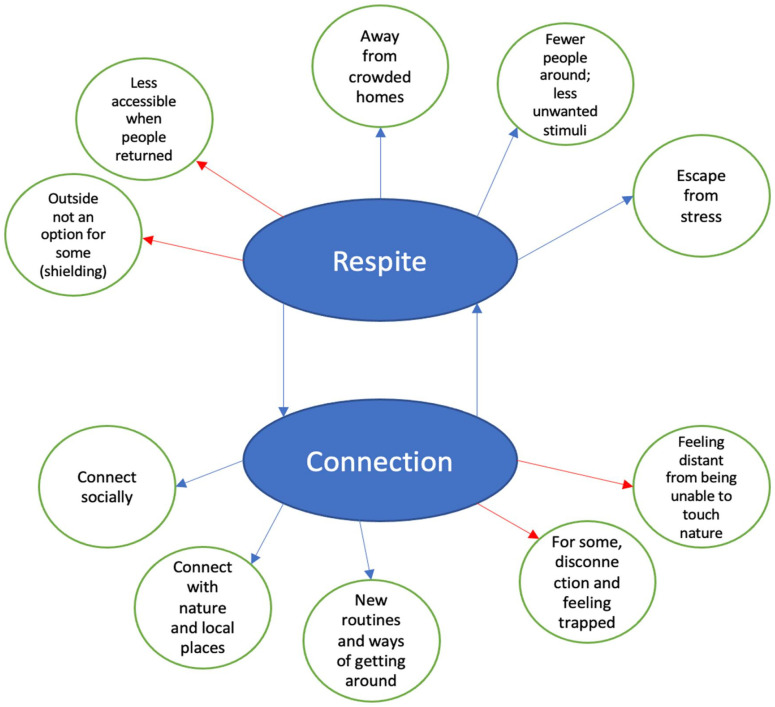
Thematic map representing two main themes and their sub-topics.

As seen in Figure 1, we analysed the responses of all 127 participants and developed 2 themes to reflect how this group of autistic people experienced nature during the Covid-19 pandemic: *respite in nature* and *connecting amid widespread disconnection.* Some autistic adults used nature quite practically during the pandemic to physically distance themselves from others or from crowded homes and to experience relief from stress. Nature also served as a way of connecting with others during a time when masks, restrictions and transmission risks made connections difficult; some participants also reported feeling more psychologically connected to nature itself during the pandemic. In the thematic map in Figure 1, blue lines indicate relationships with positive impacts while red lines indicate relationships with negative impacts. The two main themes are presented in the blue ovals, and the phrases within green circles represent sub-topics of each of those themes.

#### Theme 1: respite in nature

During the Covid-19 pandemic, many autistic participants (47.9%, *n* = 58) reported that they spent more time in nature, even as governmental restrictions limited how often people could leave their houses. These restrictions meant that some spaces were not crowded with the typical thoroughfare of traffic and commuters, allowing unprecedented experiences void of the usual triggers:

It became so much more accessible to me because there were fewer people around. I had the best time of my life in lockdown. (Woman, 45–54 years old, Scotland)

This increased accessibility had knock-on effects for some autistic adults. The absence of potentially stressful stimuli during the stricter lockdown periods allowed them to utilise nature to address both physical and mental health needs:

I was able to spend much more time outside during the first lockdown due to a complete lack of people, roadworks noise, traffic noise, venue noise etc. Thanks to this, I was able to start doing much more exercise, which in turn helped me almost eradicate the severe chronic back pain I had been suffering for a decade. I was also able to achieve the best state for my mental health I have ever had in my life due to the massive reduction in sensory overstimulations and the therapeutic nature of walking in nature. (Unknown gender, 25–34 years old, Scotland)

Nature also offered a place of respite away from crowded homes full of family members and housemates carrying out work and school from home, as well as helping to break up the workday or provide a source of movement when leaving the house for other purposes was no longer a normal part of the daily routine:

[Nature was] even more important for space and wife works at home, daughter finished school, harder to find space within the home. (Man, 45–54 years old, England)

The well-being benefits of time in nature became particularly important during the Covid-19 pandemic when life stress was more likely to be elevated. For many, nature provided a space to escape the detrimental effects of the pandemic and experience respite:

[Nature] became more a place of tranquillity when we came out of lockdown and cases of covid were still rising. (Woman, 45–54, England)

These positive experiences often did not last, however, as many participants noted that the increase in people using natural spaces as restrictions eased meant nature was no longer a feasible option for them:

Initially I appreciated that places were quieter, however when lockdowns eased places became busier than normal which increased my anxiety and reduced opportunities to engage with nature. (Non-binary person, 25–34 years old, England)

Furthermore, for some people, going outside provided the opposite of respite. For those who were shielding due to being high risk, going into nature posed a threat to their health and safety and was no longer an option:

Apart from spending a lot of time in my garden I was shielding so in the first lockdown I did not go out for a walk for months. (Woman, 55–64 years old, England)

#### Theme 2: connecting amid widespread disconnection

During the pandemic, when other ways of spending time with people were not allowed or heavily restricted, nature offered safe ways to connect – sometimes with other people, but more often with nature and with the self.

As with many people, the Covid-19 lockdown periods in the United Kingdom provided some respondents with the opportunity to develop new habits, often related to exercising outdoors. This allowed some participants to develop feelings of connection with their local natural spaces that they not previously had opportunities to explore:

Because of a lack of options for things to do during lockdown I began walking on Saturdays, exploring the local countryside, 8-10 miles each walk. This made me more appreciative of nature and its benefits to my wellbeing. (Man, 35–44 years old, England)

Similarly, the restrictions on how far people could travel and through what methods (i.e. limits on the use of public transport) meant that people shifted from their normal ways of living to adjust to a new normal. For some, this meant embracing new ways of getting around, like walking, which provided more opportunities to develop direct connections with their surroundings than if they were on a bus or train:

Now I only go where my feet can carry me. Before I would use public transport to go a bit further afield but I dont consider this a loss. In some ways i prefer it, its just me and my world, I’m not reliant on buses and trains, just on my own two feet. (Woman, 55–64 years old, England)

Perhaps because autistic adults were spending more time outside as a means of travel or exercise, many participants expressed feeling more connected to nature as a result. Whether motivated by an existing love of nature and buoyed by simply having more time or because of having nothing else to do, numerous participants described a shift in the value they placed on nature, sometimes even spurring increased pro-environmental behaviours:

I appreciate all aspects of nature more. The pandemic has made priorities clear. Also, there is a climate crisis happening that concerns me and motivates me into action, such as planting trees in our new garden. (Woman, 55–64 years old, Scotland)

Having more time to spend outside allowed some to notice nature and connect with its inhabitants. From birdwatching to butterfly counting and beyond, autistic participants described the important role that animals played in their experiences with nature:

Before I came to live at university again, I’d stay with my parents (they live in a very rural area). Every day when there was nothing else to do I would sit in the garden and count the butterflies. My mum has a huge buddleia bush and it attracts a massive amount of them – I never used to be able to name so many butterflies . . . It makes me feel productive because, when I place myself in nature, I feel like I’m relating to the animals, insects, flowers etc. I’m gaining an understanding of them and they’re gaining an understanding of me too. Like, if you stand near a buddleia bush for long enough then a butterfly will eventually land on you. And if you keep feeding the birds and the mice then they will grow comfortable with your presence – it’s so magic! (Woman, 18–24 years old, England)

Nature also provided a prompt for asynchronous social interaction, such as through social media. Solitary activities in nature did not necessarily preclude autistic people from contingent forms of social interaction. For instance, during the pandemic, one participant used their photography skills to capture elements of nature and later began sharing these pictures online, prompting knock-on effects:

[During the] lockdown I reactivated my Facebook after years and started to share my photos and got lots of positive feedback. This grew my confidence. (Woman, 35–44 years old, Scotland)

However, the introduction of Covid-19 restrictions did not always facilitate healthier connections with nature and benefits to well-being. For some, having limits on how they could engage with nature had lasting consequences:

I was, and am, very traumatised that it became illegal to walk freely, then to breathe normally, as I cannot stay indoors. I didn’t. I broke the law to be outdoors every day, and was terrified all the time that someone would force me to stop, because I would have ended my own life if that had happened. I need the wind and the sky and the water. I did not know until January this year that parents of autistic children had challenged the scope of the ban, and it had been accordingly amended to allow autistic people further travel and longer time outside. (Woman, 45–54 years old, England)

Other participants reported that the pandemic contributed to a disconnection from nature given the lack of knowledge early in the pandemic on how Covid-19 spread and what activities may pose a higher risk:

I feel more distant from nature as a result of the pandemic – I have always been very tactile in how I interact with nature – touching things, smelling them, picking them up. I trained myself not to do that at the start of the pandemic when fomite transmission was thought to be a major route of transmission, and I still haven’t got back into the habit, and I feel that it has become something of a barrier to me fully being in nature. (Non-binary person, 25–34 years old, England)

## Discussion

In this qualitative survey study of 127 autistic adults, we used reflexive thematic analysis (Braun & Clarke, 2006, 2019, 2021) to develop two thematic findings: *respite in nature* and *connecting amid widespread disconnection.*

### Theoretical framework

Ulrich et al.’s (1991) SRT served as the primary guiding theoretical framework for this research. While findings were developed inductively rather than being deductively guided by theory, we expected autistic adults to note many of the same physiological and psychological benefits that Ulrich and colleagues noted in the development of their seminal theory. Indeed, even amid the stresses of the pandemic, many autistic adults shared that spending time in nature helped them to feel calmer and less stressed, suggesting a positive relationship with well-being. This aligns with SRT’s suggestion that time in nature helps facilitate quicker recovery from stressful stimuli. Further research using the appropriate methodologies is needed to ascertain if the same physiological changes are experienced by autistic adults when in nature as those suggested in Ulrich et al.’s (1991) study. Keniger et al. (2013) pointed out the need for more causal/experimental research on the benefits of nature; this type of research may also help elucidate the specific mechanisms underpinning some of the benefits nature offers autistic people, particularly at a physiological level. However, even without this causal evidence, the stress-reduction benefits that are central to SRT aligned with the lived experiences of these autistic adults during the pandemic.

### Nature’s role during the Covid-19 pandemic

The thematic findings from this survey study are connected to literature on the topics of nature’s role during the Covid-19 pandemic and nature’s relationship with well-being more largely. While tumultuous for all, the Covid-19 pandemic had a particularly negative impact on autistic people’s well-being (Riese & Mukherjee, 2021). Alongside these challenges, some autistic adults found certain aspects of the pandemic to be pleasant, including having fewer social and sensory pressures (Hedley et al., 2021; Oomen et al., 2021). In the present survey study, autistic adults noted similarly complex feelings. The Covid-19 pandemic meant that some people could not access nature as they usually would, as seen in theme 1: *respite in nature.* Simultaneously, as also described in theme 1, many autistic adults reported that there being fewer people in public green spaces made them easier to access and that nature provided space away from interacting with people. Nature also offered a respite from crowded homes. Similarly, spending more time in nature during the pandemic helped some participants to develop stronger relationships with nature itself, reflected in theme 2: *connection amid widespread disconnection*. The role that nature played for autistic people in the Covid-19 pandemic was a complicated one, with benefits to well-being existing alongside challenges from restricted access and increased risk.

This changing relationship and increased time spent engaging with nature is not unique to this group of autistic adults. Indeed, the Office for National Statistics (2021) reported that across the United Kingdom, there was a rise in outdoor exercise during the early pandemic period. They also reported increased participation in Natural England’s People and Nature survey and improved well-being potentially associated with spending time in natural spaces. Globally, similar relationships between well-being and nature access were noted; for instance, in Japan, adults with views of green spaces reported higher self-esteem and life satisfaction, among other positive indicators of well-being (Soga et al., 2020). Alongside this increase in time outdoors and improved well-being was an increase in sedentary behaviours among both children and adults globally (Kass et al., 2021; Stockwell et al., 2021); similar variability in how autistic people experienced nature during the pandemic would be expected. For instance, as the thematic findings show, most autistic participants reported that nature had a positive relationship with well-being during this time. However, a small minority of participants reported negative experiences that should not go unnoticed. For example, six participants reported that nature was associated with increased anxiety and sensory issues. Another five participants were neutral in their responses regarding how nature impacted mental health, with responses such as ‘Not really’ and ‘I don’t think so, I like cities as well’. The inclusion of these neutral and negative perspectives helps to paint a realistic picture of the varied experiences of autistic people in nature to avoid the assumption that all autistic people will benefit from time in nature. Presumably, non-respondents would be more likely to fall into this group.

The current study raises important questions about what may be unique about how autistic people experienced nature during the pandemic. Various aspects of the pandemic, including disruption to routine, have been particularly difficult for autistic people (Davidson et al., 2021; Oomen et al., 2021). Incorporating nature experiences served as a coping mechanism for some (Davidson et al., 2021) while also forming new routines, including those around exercise, having much-needed time without social pressure or interaction, and connecting in different ways. This supports findings from Bundy et al. (2022) which suggested that routines, leisure activities and exercise were associated with decreased scores on a measure of depression for autistic adults in the United Kingdom during the pandemic. Additionally, in their qualitative analysis, the authors noted the role that both having routines and spending time in nature played in supporting behavioural regulation for their participants. It is likely this was also true to varying extents for non-autistic people; however, the impacts may be differentially important given that autistic people experienced poorer mental health during the pandemic at a higher rate than non-autistic people (Oomen et al., 2021). Based on the present study, these nature interactions likely also enabled autistic people to experience the innate well-being and physiological benefits of nature explained by Ulrich et al.’s (1991) SRT, though further research is needed to evaluate this suggestion.

Many of the findings from this study echo the experiences of 17 British adults with pre-existing conditions (none of whom disclosed being autistic) who were interviewed by Darcy et al. (2022) following the first lockdown period in summer 2020. The authors describe nature as a means of escaping from Covid-related stress, one element of theme 1: *respite in nature* from the present study. Darcy et al.’s participants also reported appreciating the sensory experience of nature, using social media and other forms of technology to engage with nature, and increasing their connection to nature during the lockdown. The similarities in lived experiences between the adults with pre-existing conditions and the autistic adults from the present study raise questions about how the experiences of these two groups are aligned and the ways they may differ. It is possible that there are elements of being autistic or having a pre-existing condition that contribute to shared or similar experiences, including being stigmatised (e.g. Rose et al., 2017; Turnock et al., 2022), having sensory needs (e.g. Thye et al., 2018; Wallis et al., 2018) or feeling socially disconnected or excluded (e.g. Jones et al., 2022; Willis et al., 2015); these similarities may be reflected in how each group interacts with and connects to nature.

Both themes developed in this study also support the idea that nature and nature-based activities were perhaps easier spaces for autistic people to be in/engage with during the pandemic as they may have allowed autistic people to mask less. Given the known detrimental impacts of masking in social situations (Cage et al., 2018; Cage, Troxell-Whitman, 2019), it is unsurprising that some autistic adults reported the pandemic period as providing a break from the need to mask (Bundy et al., 2022; Lois Mosquera et al., 2021). However, given that isolation and disconnection can be similarly harmful to well-being (e.g. Pellicano et al., 2021), opportunities to feel connection and relatedness with others over shared interests or with nature itself may have been a way of addressing the harmful impacts of social isolation in a less demanding way. Participants did not explicitly discuss the issue of inclusion in outdoor activities in the context of the Covid-19 pandemic. Rather, many described their experiences in nature as solitary and/or informal and noted that the lack of people outside made these experiences more accessible. Given the known barriers to inclusion of autistic adults in other activities (e.g. lack of support; Cameron et al., 2022), future research should investigate how effectively autistic people are included in outdoor activities both to reduce social isolation and to ensure positive experiences. While further research into the specific ways nature was related to well-being in autistic adults during the pandemic is certainly needed, the exploratory findings from the present survey study indicate that nature played an important role in meeting individual needs related to well-being.

### Strengths and limitations

This survey study, among the first to ascertain autistic adults’ views on nature’s relationship with well-being in the context of the Covid-19 pandemic, has many strengths and, as with all research, several limitations. Some of these limitations are intrinsic to online surveys (e.g. McInroy, 2016). The nature of an Internet-based survey means that participants will be only those with Internet access who are proficient at navigating websites like Qualtrics. Given that the survey took some participants up to 2 h to complete, participants will also be only those who had this spare time. Additionally, providing qualitative responses through an online survey platform might have been difficult for those participants who do not feel they express themselves most clearly in writing or who felt they did not have the time or space to do so. This method was most likely to gather the perspectives of autistic adults who were computer-literate and active in certain autistic community groups, such as those used to recruit participants. The current sample’s employment rate of nearly 40% exceeds estimates from the Office for National Statistics, which suggest that 29% of autistic adults in the United Kingdom were employed as of June 2021 (Sparkes et al., 2022). This represents only a segment of the autistic population as a result. Online surveys, particularly those that are longer, tend to have a considerable amount of drop out (Ward et al., 2017). The excellent retention of survey participants throughout most of the survey was likely related, at least in part, to the offer of a prize draw that offered 50 people a chance to win a voucher.

The location distribution is largely representative of the United Kingdom’s population breakdown given that 84.3% of the UK population live in England, 8.2% live in Scotland, 4.7% live in Wales and 2.8% live in Northern Ireland (Park, 2021). Participants were evenly spread over the 18- to 54-year age bands; however, the sample lacked higher numbers of older participants. Gathering the perspectives of a higher percentage of autistic elders (those 55 and over) would have provided valuable insight into the perspectives of this under-studied group (Robison, 2019) who may have experienced the pandemic differently to their younger counterparts. In the United Kingdom, the estimated ratio of autistic adult males to females is 3:1 (Loomes et al., 2017); the high percentage of women respondents in our study is therefore not representative of the adult autistic population. However, the gender diversity in this sample is to be expected, as biological sex and gender identity are not interchangeable concepts and transgender and gender-diverse people have higher rates of autism (Warrier et al., 2020). Additionally, the high number of women respondents is unsurprising given that researchers have anecdotally noted that recruiting for research participants online often draws more diverse autistic people (Sohn, 2019). Furthermore, the inclusion of self-diagnosed autistic adults in the present study could also explain the higher number of women who participated given that self-diagnosed autistic people are more likely to be women (e.g. McDonald, 2020).

We did not ask about race/ethnicity and sociodemographic status in the survey’s demographic questions as these data did not initially seem relevant to the research questions; further, we excluded these questions with the intention of reducing the burden on participants by asking fewer questions related to personal information. However, we acknowledge that the exclusion of this information is a limitation given the role that race/ethnicity and socioeconomic status play in nature experiences (e.g. Burnett et al., 2021). In future, we will collect this important demographic information to better ascertain the diversity of our sample.

The recruitment strategies undoubtedly influenced the specific profiles of the participants. By utilising research networks and organisational newsletters, we directly reached autistic people who were interested in contributing to research. Individuals who took part in the survey may also have been those who were more interested in nature and thus more willing to take the time to complete the survey. However, several respondents made clear that they did not enjoy nature, so their opinions influenced the themes presented here as well. Additionally, only slightly above half (52%) of the respondents said that they had a focused interest in nature. Nature, while perhaps a casual interest or hobby, was not a specific focus for a considerable number of participants, suggesting that a wider range of views were represented here. Finally, tied to perhaps the largest strength of the survey study, the process of co-creating and piloting the survey with autistic people meant that the survey was more likely to be relevant and accessible to autistic people given that it was guided by their input both topically and stylistically.

### Implications and future research

To build on the findings from this exploratory survey study of autistic adults’ experiences of nature during the Covid-19 pandemic, further research should be conducted to better understand the mechanisms that underpin these reported benefits. For instance, a close examination of what elements of nature may offer respite or be less demanding than indoor settings may help reveal why some autistic adults used nature as an escape during the pandemic. Additionally, quantitative work examining physiological markers of relaxation and stress reduction would provide further support for the application of SRT to explain some of the benefits experienced by autistic people in nature. Finally, while this survey study of 127 autistic adults helped to provide a more general picture of the experiences of some autistic adults during the pandemic, a more thorough investigation of the relationship between nature and well-being is warranted, both in the pandemic context and more broadly. This would ideally be done through semi-structured interviews with a smaller number of individuals. The aim would be to develop an understanding of their lived experiences to provide further evidence to inform policy and practice pertaining to developing autism-inclusive outdoor experiences and enable more autistic people to use nature to promote well-being.

Autistic people and their family members and caregivers may consider time in nature as a cost-effective option to address well-being-related challenges, especially those related to the Covid-19 pandemic. Becoming involved in nature-based activities that promote exercise and allow for social interaction based on shared interests may be effective for autistic people who have felt isolated during the pandemic. Autistic parents and parents of autistic children who have an interest in nature may look to outdoor activities as a means of connecting, developing memories and sharing knowledge as a family. Pursuing solo time in nature may provide respite for autistic adults feeling stressed or overwhelmed. The findings of this survey study indicate that for those autistic people who felt that nature benefitted their well-being, the options for exactly how they harnessed that benefit were varied – from long walks alone to sharing experiences with friends.

## Conclusion

Based on this survey study, nature supported well-being in autistic people during the Covid-19 pandemic in several ways. When it was not too crowded, nature was a place of respite from the stresses associated with the pandemic. Conversely, nature also offered opportunities to connect with others and with the physical environment, which was of particular importance given the widespread disconnection resulting from the pandemic.

These thematic findings are among the first to provide exploratory evidence of the role that nature played in well-being for some autistic adults during the Covid-19 pandemic according to their own perspectives. The varied experiences represented here indicate how nature was not a one-size-fits-all solution for autistic people during the unprecedented challenges of the pandemic; not all participants noted the same benefits or any benefits at all. Nature should be added to the toolbox of options for supporting well-being in autistic people as it has the potential to meet varied, sometimes conflicting, needs.

## Supplemental Material

sj-docx-1-aut-10.1177_13623613231166462 – Supplemental material for Respite and connection: Autistic adults’ reflections upon nature and well-being during the Covid-19 pandemic Click here for additional data file. Supplemental material, sj-docx-1-aut-10.1177_13623613231166462 for Respite and connection: Autistic adults’ reflections upon nature and well-being during the Covid-19 pandemic by Samantha Friedman, Roan Noble, Stephanie Archer, Jenny Gibson and Claire Hughes in Autism
