# Supplementary material for: Respite and connection: Autistic adults’ reflections upon nature and well-being during the Covid-19 pandemic
Source: Autism. 2023 Apr 27;27(8):2483–95. doi: 10.1177/13623613231166462 (PMC10140763; doi:10.1177/13623613231166462)
Supplement: sj-docx-1-aut-10.1177_13623613231166462 – Supplemental material for Respite and connection: Autistic adults’ reflections upon nature and well-being during the Covid-19 pandemic [file sj-docx-1-aut-10.1177_13623613231166462.docx]

Appendix 1 – Copy of survey

**Page 1 – participant information sheet**

Autistic perspectives on the implications of engagement with nature for sensory needs, special interests, and wellbeing

We would like to invite you to take part in a new research project. In this project, we are hoping to learn more about autistic adults’ experiences in nature. **This research is for autistic adults over the age of 18 living in the UK who are either formally diagnosed or self-diagnosed.**

Before you decide whether or not to take part in our survey, please read the information below:

**Who is conducting and funding this research?**

- This research is being conducted by **[researcher]**, a [role] in the [research centre and department] at the [authors’ university].
- [researcher] is supervised by **[researchers]**.
- The study has been reviewed by the [authors’ university/departmental] Research Ethics Committee and is funded by the [department].

**Purpose of the study**

- The purpose of this study is to **gather the perspectives of autistic adults about their experiences of nature**.
- The survey focuses upon general information including:
  - your thoughts and feelings about being in nature
  - sensory profile
  - childhood experiences in nature
  - experiences in nature as an adult, including during the Covid-19 pandemic
  - special interests

**Why have I been chosen?**

- You are being asked to take part in this survey because **you are an autistic adult over the age of 18** who lives in the United Kingdom.

**What would happen if I take part? What do I have to do?**

- Taking part in the research involves completing an online survey.
- To view the survey questions ahead of time, click here: [link]
- There are around 20 questions in the survey.
  - The format of the questions varies:
    - Some of the questions are multiple choice
    - Others provide text boxes for you to explain your answer. You can write as much or as little as you’d like.
- You can leave the survey and return to complete it later; you do not have to finish it all in one sitting.
- You can skip any questions in the survey, though it’s helpful if you try to answer as many as you can. All of your answers will be anonymous.

**Do I have to take part?**

- You do not have to take part.
- You can take time to decide if you would like to complete the survey.
- You can also contact [researcher] at [email] if you have any questions.

**Are there possible risks and benefits of taking part?**

- There are few to no risks to taking part. The only possible downside to taking part is the time needed to complete the survey.
- If you’re happy to provide your email at the end of the survey, you will be entered in a drawing for one of 50 £10 Amazon vouchers to thank you for taking part. Your email will be separated from your responses, meaning that your responses will be anonymous; your email will be deleted after the voucher draw has taken place and prizes have been sent.

**What happens to the results of the research?**

- Results will be presented at conferences and written up in academic journals, ideally within the next two years. Results are normally presented in terms of groups of individuals. If any individual data are presented, the data will be totally anonymous, without any means of identifying the individuals involved.
- Additionally, results will be included in one of the researcher’s [degree].
- The research team will make available a summary of the findings on social media following data analysis. On the final page of the survey, you can also opt in to receiving an email with a summary of the study’s findings.

**How will my information be stored?**

- The information will only be used by the research team and will be deleted at the end of the study.
- If you provide an email at the end of the survey, your email will be separated from your responses and deleted following completion of whichever actions to which you consent (e.g., voucher draw, receiving study summary, and/or saving email address for two years to participate in future interviews).
- Data storage follows the DPA 2018 guidance; for more information please see: [link]

**What should I do next?**

If you’d like to participate in the survey, please continue on to the consent form by clicking the button on the bottom right of your screen. Please read and complete the consent form; you’ll then be able to continue on to the survey.

**Thank you very much for taking the time to read this information and for considering participating. If you have any questions, please feel free to contact [researcher] [email].**

**Page 2 – consent form**

Consent must be given on all items to continue with this survey.

1. I confirm that I am an autistic adult (aged 18 or above) who was either self-diagnosed or professionally diagnosed. ___Yes ___No

2. I have read the information sheet about the research. ___Yes ___No

3. I understand that my participation in this survey is voluntary, and I can leave the survey and return to complete it at any time or leave questions blank. ___Yes ___No

4. I understand that I can provide an email address to enter a drawing for a voucher, participate in potential future interviews, and/or receive a summary of the study’s findings. My email address will immediately be separated from my responses and permanently deleted once the action to which I consent has been completed. My responses will be anonymous and stored in line with the Data Protection Act 2018. ___Yes ___No

5. I understand that because my responses will be anonymous, researchers cannot identify my data and so it is not possible for my responses to be deleted after I submit the survey. ___Yes ___No

6. I understand that I can contact [researcher] [email] at any time to discuss this research. ___Yes ___No

**What do we ask about in this survey?**

**Demographic and personal information**

This section will provide us with general information about you.

**Nature use**

This section asks about the amount of time you spend in nature and in what ways you spend time in nature. This will allow us to understand how much of a role nature plays in your life.

**Adult nature experiences**

This section focuses on experiences you’ve had with or in nature as an adult. We will also ask several questions about your connection to nature. This will allow us to better understand how nature impacts (or does not impact) you personally as well as your opinions on the meaning of the term ‘connection to nature.’

**Childhood nature experiences**

This section focuses on the experiences that you had in nature as a child. This will allow us to understand what influences might have shaped your current feelings about nature and the experiences you’ve gone on to have with nature as an adult. This is also important for developing supportive and accessible outdoor programmes for autistic children, particularly at school.

**Note on language:**

For this survey, nature is defined as anything in the physical world including outdoor green spaces, animals, other landscape features like mountains and rivers, and plants.

Spending time or being **IN** nature refers to being outside amongst features of the physical world, including green spaces, animals, and plants. This includes interacting with nature by touching it or using natural materials as well as sitting, walking, or otherwise moving in natural spaces.

Your responses will not be submitted to researchers until you reach the last page of the survey and click the final arrow button. There is a message on the last page to inform you that you are about to submit your responses. Prior to submitting, you can move throughout the survey using the arrow buttons to edit or return to responses as you’d like.

**Page 3 – demographic and personal information**

1. What is your gender?

- Woman
- Man
- Non-binary
- Prefer not to answer
- Other: (free response space)

2. What is your age?

- 18-24 years
- 25-34 years
- 35-44 years
- 45-54 years
- 55-64 years
- 65-74 years
- 75 years or older
- Prefer not to answer

3. What is your employment status?

- Employed in a full-time job
- Employed in a part-time job
- Not currently employed but looking for work
- Not currently employed and not looking for work
- Student
- Retired
- Unable to work
- None of these describe my employment status
- Prefer not to answer

4. Where in the UK do you live?

- England
- Scotland
- Wales
- Northern Ireland
- Prefer not to answer

5. Do you have any specific accessibility needs (physical or otherwise) that may affect how often you leave home and where you go? (e.g., hypermobility, use of mobility aid, use of assistive technology)

- Yes
- No
- Prefer not to answer

*(only display next question if ‘Yes’ is selected)*

5.1 If you’d like to provide more information about your specific physical or accessibility needs, please use this text box. You do not have to explain or provide more information if you don’t want to.

(open text box)

6. Special interests are focused interests or passions in particular topics or activities that bring joy, comfort, or other positive benefits to your life and last for multiple years. Hyperfixations are similar to special interests, however they are shorter-term and last anywhere from several days to just one or two years. They are common in autistic people who also have ADHD.

Do you have any current special interests and/or hyperfixations?

- Yes, I have one or both
- No, I have neither
- Prefer not to answer

*(only display next question if ‘Yes’ is selected)*

6.1 If yes, please describe your special interest(s) and/or hyperfixation(s).

(open text box)

6.2 *(only display the following question if ‘Yes’ was selected to question 6)* Are any of your hyperfixations and/or special interests related to nature?

- Yes
- No

6.3 *(only display if ‘Yes’ is selected)* In what way(s) is/are your hyperfixations and/or special interest(s) related to nature? How does being in nature support your hyperfixations and/or special interest(s)?

(open text box)

**Page 4 – nature use**

7. Which of the following best describes the area you live in currently?

- An urban area (in a city)
- A suburban area (not in a city, but with other houses, roads, and shops around)
- A rural area (not many other houses or shops around, mostly open space)

8. Which of the following best describes the outdoor green spaces you have access to daily? ‘Access’ means that you could easily visit these spaces if you wanted to do so. ‘Green spaces’ include forests, fields, pitches, parks, and other open spaces with grass or trees.

- I have access to several different outdoor green spaces.
- I have access to only one outdoor green space.
- I have outdoor green spaces nearby, but physical limitations prevent me from visiting.
- I have outdoor green spaces nearby, but there’s another reason I’m not able to easily access them.
- I don’t have any outdoor green spaces nearby

9. How much time per week do you spend outside doing any activity (e.g., walking, physically interacting with nature, sitting, exercising, using bike or scooter)?

- Less than 1 hour per week
- More than 1 hour per week but less than 3 hours
- More than 3 hours per week but less than 5 hours
- More than 5 hours per week

**Page 5 – adult nature experiences**

10. Did the Covid pandemic and lockdowns change the amount of time you spent in nature?

- Yes, I spent more time in nature
- Yes, I spent less time in nature
- No, I spent the same amount of time in nature as usual

11. Did the Covid pandemic and lockdowns change your relationship with nature?

- Yes
- No

11.1 If yes, how did your relationship with nature change because of the Covid pandemic and lockdowns? If no, leave this box blank.

(open text box)

12. Does being in nature have an impact on your mental health? If yes, please describe the impact that it has. If no, please describe other activities or environments that you feel affect your mental health.

(open text box)

12.1 Does being unable to access nature have an impact on your mental health? If so, how?

(open text box)

13. Does being in nature change your sensory needs or preferences? If so, please explain how. For instance, are you more able to tolerate certain foods when cooking around a campfire? Are you more able to tolerate certain textures if made of natural material?

(open text box)

14. If you want to share any additional information about your personal sensory needs, please use this text box. Examples of information you might want to provide includes:

-tools you use to self-regulate

-items that help you in daily life (e.g., sunglasses, ear defenders)

-situations you seek out or aim to avoid because of sensory stimuli

(open text box)

15. What does the term ‘connection to nature’ mean to you? There are no right or wrong answers. Answer based on your personal knowledge and experience.

(open text box)

16. Short Form Version of the Nature Relatedness Scale (NR-6)

Instructions: For each of the following, please rate the extent to which you agree with each statement, using the scale from 1 to 5 as shown below. Please respond as you really feel, rather than how you think “most people” feel.

1. My ideal vacation spot would be a remote, wilderness area.
2. I always think about how my actions affect the environment.
3. My connection to nature and the environment is a part of my spirituality.
4. I take notice of wildlife wherever I am.
5. My relationship to nature is an important part of who I am.
6. I feel very connected to all living things and the earth.

Answer choices:

1= disagree strongly

2= disagree a little

3= neither agree or disagree

4= agree a little

5= agree strongly

17. Were there any items from question 16 that you felt were confusing, unclear, or otherwise problematic? If yes, which item(s)? You can simply refer to the statement number (1-6). You can provide an explanation of your thoughts on the item if you’d like.

(open text box)

**Page 6 – childhood nature experiences**

The following questions ask about your childhood. Talking about childhood can be upsetting for some. You don't have to share anything you feel is too personal. Please only share what you are comfortable sharing. Please feel free to take a break or leave questions blank if needed.

18. Please describe the experiences in nature you had during your childhood. Here are a few questions you may want to think about. You do not have to answer all or any of these questions, though.

-How much time did you spend outside as a child?

-Did you have any important experiences in nature as a child? If so, what?

-Did you enjoy being outside as a child? If so, what specifically did you enjoy doing?

-Did you spend time outside with your family as a child? If so, what did you do with your family while outside?

-Did you participate in any outdoor groups such as Scouts?

(open text box)

19. Do you wish anything was different about your experiences in nature as a child? If yes, what do you wish was different?

(open text box)

20. If there is any feedback you’d like to share about the survey, please tell us here.

(open text box)

**Page 7 – email address**

If you’re willing, it would be helpful for us to know the first part of your postcode. This will allow us to see how close our participants are to greenspaces. Are you willing to share the first part of your postcode? For example, if your postcode is AB1 CD2, you’d share ‘AB1.’

(open text response)

Thank you very much for taking the time to complete this survey!

You have the choice to provide your email address below for three different reasons. You can choose to give your consent for one, two, three, or none of these reasons. The reasons are:

- To be entered in a prize draw for one of 50 £10 Amazon vouchers
- To possibly be interviewed in the future
  - We may wish to expand on this work in the future by interviewing autistic adults about their experiences with nature
- To receive an email with a summary of the findings once we complete data analysis

Your email address will be separated from your responses after you submit your survey. Your email address will be deleted after the completion of whichever actions you consent to.

Would you like to be entered in the prize draw?

- Yes
- No

Can we save your email to contact you in the future about doing an interview? If you say yes, email addresses will be deleted after two years.

- Yes
- No

Would you like to receive a summary of the study’s findings via email?

- Yes
- No

This is the end of the survey. Clicking the arrow at the bottom of the page will complete the survey and submit your responses to the researchers. After submitting, you won't be able to go back or re-enter the survey. If you’d like to make changes or review any of your responses, please use the ‘back’ arrows to revisit earlier pages of the survey.

If you have any questions about the study, please email [researcher] at [email]. Have a great day!
